# Intravitreal Aflibercept Outcomes in Patients with Persistent Macular Exudate Previously Treated with Bevacizumab and/or Ranibizumab for Neovascular Age-Related Macular Degeneration

**DOI:** 10.1155/2014/497178

**Published:** 2014-11-20

**Authors:** David R. Griffin, Preston P. Richmond, John C. Olson

**Affiliations:** ^1^The University of Central Florida College of Medicine, 6850 Lake Nona Boulevard, Orlando, FL 32827, USA; ^2^Central Florida Retina and the Macular Degeneration Center, 44 Lake Beauty Drive, Suite 300, Orlando, FL 32806, USA

## Abstract

*Purpose*. To assess whether intravitreal aflibercept (2.0 mg) can effectively reduce persistent macular exudate and enhance visual acuity in ranibizumab (0.5 mg) and/or bevacizumab (1.25 mg) treatment resistant patients with neovascular age-related macular degeneration. *Methods*. This retrospective study included 47 treatment resistant eyes from 47 patients switched to intravitreal aflibercept injections after receiving a minimum of 3 injections with either ranibizumab or bevacizumab. Snellen visual acuity and optical coherence tomography were assessed just prior to the first injection (baseline) and prior to the fourth injection (final). Additionally, anatomical regions of persistent macular exudate were tracked to determine if these areas yielded varying responses to aflibercept. *Results*. At baseline, patients had received an average of 11.3 injections with any prior anti-VEGF drug (SD 5.96). For whole group analysis, baseline and final central retinal thickness were 370.57 *µ*m and 295.7 *µ*m (*P* ≤ .001), respectively. Baseline and final retinal fluid volumes were 4.81 mm^3^ and 4.37 mm^3^ (*P* ≤ .001), respectively. Baseline and final logMAR were 0.56 and 0.53 (*P* = 0.301), respectively. Anatomic location of persistent exudate did not appreciably alter treatment outcome. *Conclusion*. Central retinal thickness and total retinal fluid volume were reduced in ranibizumab and/or bevacizumab treatment resistant patients following three aflibercept injections. No appreciable change in visual acuity was noted.

## 1. Introduction

Age-related macular degeneration, or AMD, is a leading cause of blindness in America, as well as industrialized countries worldwide, and it is estimated that nearly 10 million Americans suffer from AMD [1, 2]. Until recently there have been two leading treatments for neovascular AMD which inhibit vascular endothelial growth factor (VEGF) in order to diminish choroidal neovascularization (CNV), namely, ranibizumab (Lucentis, Genentech, Inc., South San Francisco, CA) and the off-label AMD therapeutic bevacizumab (Avastin, Genentech, Inc., South San Francisco, CA). Both drug therapies have been shown to effectively reduce vision loss and potentially improve vision in many AMD patients [3, 4]. Many studies have confirmed the outcomes and risks of using either of these drugs for the treatment of neovascular AMD and a majority of these studies, including multicenter comparative clinical trials such as the CATT, MANTA, GEFAL, and IVAN studies, have shown little to no difference in outcomes of patients that are treated with either drug [5–12]. Therefore, today both ranibizumab and bevacizumab are recognized as efficacious therapeutics for the treatment of neovascular AMD. Despite widely accepted use of either drug, there remain many patients that display persistent macular exudation posttreatment.

In November 2011, another inhibitory VEGF drug was approved by the FDA for treatment of neovascular AMD, namely, aflibercept (Eylea; Regeneron, Tarrytown, New York, USA, and Bayer, Berlin, Germany). Aflibercept has been shown to have a significantly higher binding affinity for VEGF than either bevacizumab or ranibizumab [13]. Theoretical calculations also suggest that a single aflibercept intravitreal injection would last between 48 and 83 days as compared to only 30 days for a ranibizumab injection [14]. In phase III trials aflibercept was shown to be noninferior to ranibizumab treatments and elicited the same outcome as a monthly ranibizumab injection regimen when administered bimonthly following 3 monthly injections over a 52-week period [14, 15]. Consequently, aflibercept is being introduced as a noninferior treatment option that requires fewer injections and is more affordable for patients.

Recently, a handful of studies have investigated the effects of aflibercept on patients resistant to either bevacizumab or ranibizumab therapy. Results from these studies are varied with some reporting increased visual acuity (VA) following aflibercept treatment in patients with persistent exudation, as well as no change in VA posttreatment [16–19]. This current study seeks to further define the clinical and anatomical outcomes of aflibercept therapy in the previously treatment resistant neovascular AMD population. This study also seeks to determine if there is an association between anatomic location of persistent choroidal exudation and response to intravitreal aflibercept treatment in this same treatment resistant population which is yet to be thoroughly discussed in the literature.

## 2. Methods

Institutional review board approval was obtained from the University of Central Florida. All patients selected for this retrospective study were a subset of patients that had previously qualified for the treatment of neovascular AMD and were at least 55 years old. Inclusion criteria required that patients (1) had to have been initially treated with either bevacizumab or ranibizumab for the treatment of neovascular AMD with a minimum of three intravitreal injections of either drug, (2) had to be considered treatment resistant, excluding partial responders that displayed persistent choroidal exudation while receiving initial anti-VEGF therapy with either bevacizumab or ranibizumab, and (3) had to have received a baseline visit that was recorded, being the visit immediately prior to conversion to aflibercept therapy. Patients were excluded from the study if (1) the OCT was dry at any time during the three injections prior to conversion to aflibercept, (2) elapsed time between prior treatment and the switch to aflibercept exceeded 63 days, (3) following conversion to aflibercept therapy the patient interrupted consecutive aflibercept treatment with an alternative anti-VEGF therapy or any other intervention for the treatment of AMD, and (4) they did not have at least three aflibercept injections recorded after conversion.

Criteria for patient improvement during treatment with bevacizumab and/or ranibizumab, and later with aflibercept therapy, were determined by both optical coherence tomography (OCT) and Snellen VA measurements. OCT images were obtained using the Heidelberg Spectralis HRA+OCT 5.3.3.0 (Heidelberg Engineering, Inc., Vista, CA). Proprietary algorithms, active eye tracking (TruTrack), and AutoRescan were used to both accurately measure macular volume and determine CRT. These features also allowed accurate comparison of scans for a single patient using point-to-point correspondence between scans. Macular volume as measured by OCT was defined as total retinal fluid in this study. Also, retinal scans were individually examined to determine the anatomic location of fluid accumulation and subsequently defined as intraretinal fluid (IRF), subretinal fluid (SRF), or multiple layer fluid (MLF). VA was also collected with use of a Snellen chart using best corrected visual acuity (BCVA). For statistical purposes, Snellen VA values were converted into logMAR. OCT and VA results reported and analyzed in this study include those values obtained just prior to aflibercept treatment (baseline) as well as those obtained just prior to the 4th aflibercept injection (final). Other qualitative analyses such as slit lamp and fundus examination were conducted.

At the initial patient encounter OCT and VA were conducted to determine the status of the macula and patient vision. Following a minimum of three injections of bevacizumab and/or ranibizumab, physicians changed patients to aflibercept injections if there was persistent macular exudation. Following conversion to aflibercept therapy, OCT and VA measurements continued to be obtained at every visit up until just prior to the 4th aflibercept injection. These measurements were first analyzed for the entire patient sample which we refer to as whole group analysis. Following whole group analysis, patients were further divided into subgroups according to the anatomic location of persistent macular exudation mentioned earlier in order to determine whether any of these regions had varying responses to intravitreal aflibercept therapy. Each subgroup was assessed using the same methods as the whole group analysis.

Intravitreal injections were given to patients according to Central Florida Retina and the Macular Degeneration Center protocol. Topical anesthetic was applied and a sterile cotton swab was soaked in sterile 4% lidocaine and applied numerous times to the area receiving the injection. Patients were situated with a sterile lid speculum after which they were given drops of povidone-iodine (5%) applied at least three times. Injections were given using a 1 mL tuberculin syringe with a 30-gauge needle. All injection doses for bevacizumab and ranibizumab were 1.25 mg and 0.5 mg, respectively. Aflibercept injections were given at a dose of 2 mg.

Within-subject comparisons of continuous variables were conducted using Wilcoxon signed ranks tests. *P* values < 0.05 were considered statistically significant. Statistical analyses were conducted using SPSS 20.0 (IBM; Chicago, IL). Power analyses were conducted using G^*^Power 3.1.3 [20].

## 3. Results

A total of 58 patients with persistent retinal fluid following treatment with either bevacizumab or ranibizumab were switched to 2.0 mg aflibercept injections. Of these patients, 47 eyes (27 OD) from 47 patients met the inclusion criteria described previously. Of this sample, 20 patients were male and 27 female and average age was 80.5 years with a range of 59–98 years (Table 1). Prior to aflibercept treatment, this patient sample included 15 patients with exclusive bevacizumab therapy which received an average of 10 injections (SD 5.29, range 3–22), 14 patients with exclusive ranibizumab treatment with an average of 11.14 injections (SD 7.14, range 3–26), and 18 patients that had received an average of 12.5 injections of either drug (SD 5.57, range 8–27). As a combined group, regardless of injection history, this patient sample had received an average of 11.3 injections with any prior anti-VEGF drug (SD 5.96, range 3–27). The average interval between the last injection with a prior anti-VEGF drug and the first aflibercept injection was 42.9 days (SD 1.9, range 27–63) (Table 2).

Patients were first analyzed as an entire group in regard to CRT, total retinal fluid volume, and VA (logMAR). Mean CRT for the whole group analysis decreased by 74.9 *μ*m with a baseline and final CRT of 370.57 *μ*m (IQR 280.5–428.5) and 295.7 *μ*m (IQR 232–335.5, *P* ≤ .001), respectively. Additionally, mean total retinal fluid decreased by 0.44 mm^3^ following 3 aflibercept injections with a baseline and final fluid volume of 4.81 mm^3^ (IQR 3.06–7.6) and 4.37 mm^3^ (IQR 2.82–7.18, *P* ≤ .001), respectively. Baseline and final logMAR for the group were 0.56 (IQR 0.29–0.99) or 20/73 and 0.53 (IQR 0.24–0.71, *P* = 0.301) or 20/67, respectively (Table 3). Following whole group analysis subgroups were analyzed.

Subgroup analysis revealed that, regardless of the anatomic location of retinal fluid, there were statistically significant reductions in both CRT and total retinal fluid for all subgroups. Seven patients (14.9%) were found to have persistent IRF. These patients had a baseline and final CRT of 422.29 *μ*m (IQR 340.5–491.5) and 300.57 *μ*m (IQR 281–338, *P* = 0.018), respectively. Baseline and final fluid volumes for the IRF subgroup were 5.19 mm^3^ (IQR 3.34–6.63) and 4.46 mm^3^ (IQR 2.91–5.25, *P* = 0.018) and baseline and final logMAR were 0.90 (IQR 0.65–1.3) or 20/157 and 0.86 (IQR 0.51–1.02, *P* = 0.596) or 20/146, respectively. 27 patients (57.4%) were classified as having persistent SRF. These patients had a baseline and final CRT of 379.33 *μ*m (IQR 294.5–417) and 309.48 *μ*m (IQR 242.5–344, *P* = 0.001), respectively. Baseline and final fluid volumes were 4.62 mm^3^ (IQR 3.03–6.79) and 4.25 mm^3^ (IQR 2.81–5.39, *P* = 0.007) and baseline and final logMAR were 0.47 (IQR 0.27–0.63) or 20/59 and 0.45 (IQR 0.21–0.57, *P* = 0.692) or 20/56, respectively. Finally, 9 patients (19.1%) were determined to have persistent multiple layer fluid persistence. These patients had a baseline and final CRT of 360.33 *μ*m (IQR 271–425) and 278 *μ*m (IQR 232–307, *P* = 0.021), respectively. Baseline and final fluid volumes were 5.40 mm^3^ (IQR 3.52–7.6) and 4.83 mm^3^ (IQR 2.88–7.58, *P* = 0.008) and baseline and final logMAR were 0.48 (IQR 0.3–0.58) or 20/60 and 0.48 (IQR 0.28–0.52, *P* = 0.933) or 20/60, respectively (Table 4).

**Figure 1:**
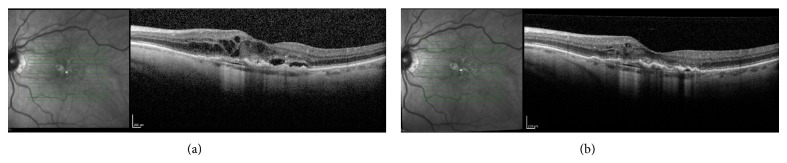
A patient with AMD previously treated with 14 ranibizumab injections and 13 bevacizumab injections shows persistent multilayer fluid on OCT (a). Following three aflibercept injections (b) there was marked reduction in total fluid volume as well as a reduced CRT, 3.43 to 3.07 mm^3^ and 405 to 293 *μ*m, respectively.

**Figure 2:**
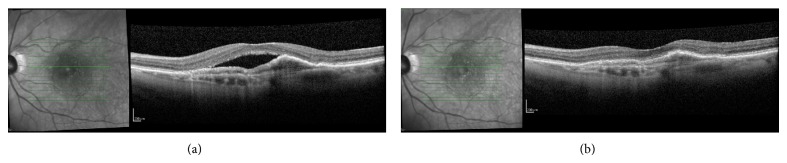
A patient with AMD previously treated with 4 intravitreal ranibizumab injections shows persistent subretinal retinal fluid on OCT (a). Following one intravitreal aflibercept injection (b) there was marked reduction of subretinal fluid and reduced CRT, 7.61 to 6.88 mm^3^ and 442 to 223 *μ*m, respectively.

The greatest reduction in CRT and total fluid volume were 121.72 *μ*m and 0.73 mm^3^, respectively, both of which were seen in the IRF subgroup. VA findings were consistent with those of the whole group analysis, showing either no change in VA or slight increases of just more than 1-2 letters on a Snellen chart. Again, these were not statistically significant. Following three aflibercept injections whole group analysis showed that 77% of patients experienced both a reduced CRT and total retinal fluid volume (Figure 1). Many of these patients showed changes following one aflibercept injection (Figure 2).

## 4. Discussion

The treatment of neovascular AMD continues to progress over time and the therapeutic options are becoming more defined. This study indicates that an improved anatomical outcome can be achieved in patients with persistent macular exudation despite prior treatment with either ranibizumab or bevacizumab following 3 aflibercept injections. This study also indicates that, despite the anatomic region of persistent macular exudation, CRT decreases in previously treatment resistant patients when switched to aflibercept, something that has not been explored in depth in the current literature. In both the whole group and subgroup analysis there was no clinically or statistically significant change in VA following three aflibercept injections.

Even though aflibercept was able to resolve persistent macular exudate in our patient sample it remains controversial if a dry macula after anti-VEGF treatment allows for regained VA. It may be that these treatment resistant patients need more time once therapy has resolved macular exudate before VA begins to improve. It was demonstrated in the VIEW I and II trials that while aflibercept did show resolution of macular exudation in treatment naïve eyes earlier than ranibizumab, there were no obvious gains in VA between therapeutics after one year [15, 21, 22]. Moreover, patients in this study received all three aflibercept injections and had all visits recorded prior to an elapsed time of one full year without appreciable increases in VA, which might suggest that indeed more time is needed for an improved VA to become apparent. At the conclusion of three aflibercept injections the nonstatistically significant change in VA was a mean increase of 0.03 logMAR, equivalent to just slightly more than a one letter gain on a Snellen chart. Similar insignificant findings were also found to be the case for all subgroups evaluated for changes in VA following aflibercept treatment. It may be possible that neural damage secondary to AMD is extensive enough in these patients that no appreciable VA will return.

The favorable response of patients with persistent exudation to aflibercept treatment may reasonably be attributed to a number of explanations, including the molecular differences that exist between anti-VEGF therapeutics. Bevacizumab is a full length, humanized, recombinant monoclonal antibody whereas ranibizumab is an affinity matured, humanized, monoclonal antibody Fab fragment [23]. The original development of these two anti-VEGF drugs was aimed at slightly different therapeutic approaches. Bevacizumab was designed with the intent to treat systemic, advanced cancers whereas ranibizumab was designed as a Fab fragment with the intent to allow its 49 kD size to penetrate the 79 kD retinal exclusion limit [24]. Yet despite these subtle differences their outcomes have been shown in many multicenter trials to be near equivalent as both bevacizumab and ranibizumab effectively target and inhibit the receptor binding domains of all VEGF-A isoforms. In contrast, aflibercept is a soluble decoy fusion protein capable of binding all VEGF-A and VEGF-B isoforms, as well as placental growth factor, with greater affinity than other anti-VEGF therapeutics currently available [24, 25]. Also, as discussed earlier, theoretical calculations have estimated that a single aflibercept intravitreal injection lasts between 48 and 83 days as compared to only 30 days for a ranibizumab injection [10]. Although intraocular half-life of aflibercept in humans has not been confirmed, these calculations, in addition to aflibercept's stronger binding affinity and broader selectivity, offer a reasonable explanation as to how it has been efficacious in reducing both CRT and total fluid volume in the treatment resistant macula. Another alternative may be that tachyphylaxis occurs due to the nature of frequent dosing with bevacizumab or ranibizumab. It is important to note that some patients in this study had received as many as 27 injections of any anti-VEGF therapeutic before the switch to aflibercept. Also, the question remains as to whether patients that were once treatment resistant and who are now currently treated with aflibercept and experiencing improved anatomical outcomes will eventually become tachyphylactic to aflibercept.

Results from this study, and others, raise the important question regarding the need to justify time consuming and costly treatment with anti-VEGF therapy, particularly in nonresponders converted to aflibercept, when there is improved macular exudate but no gains in VA. While it is true that there was no statistically significant improvement in VA it is important to note that there was also no statistical decline in VA. Anti-VEGF therapeutics, even when treatment resistant eyes are switched to aflibercept, still serve the purpose of bringing disease progression to a standstill in most patients with neovascular AMD. The potential for aflibercept to restore VA in treatment resistant eyes is also currently under much investigation and while this study shows no improvement in VA, others have found increased visual outcomes for treatment resistant patients switched to aflibercept [16]. As discussed earlier, it is critical to further understand if patients need more time with aflibercept treatment before a gain in VA is noted. One thing seems sure: more research is necessary in order to come to any certain conclusion regarding the need for aflibercept treatment in ranibizumab and/or bevacizumab nonresponders.

While our evaluation of persistent macular exudate by anatomic region did reveal that CRT is reduced in response to aflibercept despite anatomic region in all subgroups it is important to recognize that our patient subgroups were small due to inclusion criteria. Future studies might evaluate larger groups of patients with specific retinal fluid lesions, including intraretinal, subretinal, and subpigment epithelial fluid persistence to further define the results of this study. Results concerning VA for these same subgroups may be further defined with an increased sample size.

Limitations of this study include a small sample size, longer than desired time period between converting to aflibercept from prior anti-VEGF therapy, retrospective design, lack of a control arm, time span of only three aflibercept injections, and the use of Snellen VA. Ideally, time of converting from a previous anti-VEGF to aflibercept would have been no more than an average of 30 days with exclusion criteria set at maximum 45 days. We chose to extend our exclusion criterion to 63 days in order to have a sufficient population to ensure reliable data. As a retrospective, uncontrolled study assessing a patient population treated by multiple retinal specialists there inherently are constraints on data interpretation. Although this study did not include additional injections beyond three aflibercept treatments the original intent was to see if there were marked changes in VA or macular exudate in the immediate period of aflibercept treatment. While this study indicates that there was no appreciable change in VA, some caution should be taken in regard to VA analysis as it has been shown that Early Treatment Diabetic Retinopathy Study (ETDRS) charts are more accurate in assessing VA than Snellen charts, particularly with poor VA populations [26].

In conclusion, this research study shows that following three intravitreal aflibercept injections in exudate persistent neovascular AMD patients there is marked reduction of CRT and total retinal fluid volume as seen on OCT, with a majority of patients (77%) having a favorable anatomic response. This study also shows that both a reduced CRT and total retinal fluid volume can be achieved in exudate persistent patients regardless of the anatomic location of exudation in the retina, specifically IRF, SRF, and MLF. No clinically or statistically significant changes in VA were noted.

## Figures and Tables

**Table 1:** Baseline characteristics.

Total patients, *n*	47
Total eyes, *n* (%)	47
Male, *n* (%)	20 (42.6%)
Right eye, *n* (%)	27 (57.4%)
Age, years (±SD, range)	80.5 (±8.02, 59–98)

**Table 2:** Injection history prior to aflibercept conversion.

Ranibizumab	
Patients treated previously with only ranibizumab, *n* (%)	14 (29.8%)
Prior injections, mean (±SD, range)	11.14 (±7.14, 3–26)
Bevacizumab	
Patients treated previously with only bevacizumab, *n* (%)	15 (31.9%)
Prior injections, mean (±SD, range)	10 (±5.29, 3–22)
Both	
Patients treated previously with both, *n* (%)	18 (38.3%)
Prior injections, mean (±SD, range)	12.5 (±5.57, 8–27)
All patients	
Prior injections, mean (±SD, range)	11.3 (±5.96, 3–27)
Interval between 1st IVA and previous anti-VEGF, days (±SD, range)	42.9 (±1.9, 27–63)

IVA, intravitreal aflibercept.

**Table 3:** Whole group anatomical and visual measurements following aflibercept.

	Baseline mean (IQR)	Final mean (IQR)	*P* value
Central retinal thickness (*µ*m)	370.57 (280.5–428.5)	295.7 (232–335.5)	<0.001
Visual acuity (logMAR)	0.56 (0.29–0.99)	0.53 (0.24–0.71)	0.301
Total retinal fluid (mm^3^)	4.81 (3.06–7.6)	4.37 (2.82–7.18)	<0.001

logMAR, logarithm of minimum angle of resolution.

IQR, interquartile range.

**Table 4:** Subgroup anatomical and visual measurements following aflibercept.

	Baseline mean (IQR)	Final mean (IQR)	*P* value
Persistent intraretinal fluid^†^			
Central retinal thickness (*µ*m)	422.29 (340.5–491.5)	300.57 (281–338)	0.018
Visual acuity (logMAR)	0.90 (0.65–1.3)	0.86 (0.51–1.02)	0.596
Total retinal fluid (mm^3^)	5.19 (3.34–6.63)	4.46 (2.91–5.25)	0.018
Persistent subretinal fluid^‡^			
Central retinal thickness (*µ*m)	379.33 (294.5–417)	309.48 (242.5–344)	<0.001
Visual acuity (logMAR)	0.47 (0.27–0.63)	0.45 (0.21–0.57)	0.692
Total retinal fluid (mm^3^)	4.62 (3.03–6.79)	4.25 (2.81–5.39)	0.007
Persistent multiple layer fluid^§^			
Central retinal thickness (*µ*m)	360.33 (271–425)	278 (232–307)	0.021
Visual acuity (logMAR)	0.48 (0.3–0.58)	0.48 (0.28–0.52)	0.933
Total retinal fluid (mm^3^)	5.40 (3.52–7.6)	4.83 (2.88–7.58)	0.008

^†^7 patients, ^‡^27 patients, and ^§^9 patients.

logMAR, logarithm of minimum angle of resolution.

IQR, interquartile range.
